# A Meta-Analysis of miR-499 rs3746444 Polymorphism for Cancer Risk of Different Systems: Evidence From 65 Case-Control Studies

**DOI:** 10.3389/fphys.2018.00737

**Published:** 2018-06-12

**Authors:** Xianglin Yang, Xuelian Li, Baosen Zhou

**Affiliations:** ^1^Department of Clinical Epidemiology, First Affiliated Hospital, China Medical University, Shenyang, China; ^2^Department of Epidemiology, School of Public Health, China Medical University, Shenyang, China; ^3^Key Laboratory of Cancer Etiology and Prevention, Liaoning Provincial Department of Education, China Medical University, Shenyang, China

**Keywords:** miR-499, rs3746444, cancer risk, polymorphism, meta-analysis

## Abstract

MicroRNAs (miRNAs) are a class of endogenous, short and non-coding RNAs that may play important roles in the pathogenesis of tumor. The associations between microRNA-499 rs3746444 polymorphism and cancer risk in different systems remain inconclusive. This article is aimed to obtain more exact estimation of these relationships through a meta-analysis based on 52,456 individuals. We retrieved relevant and eligible studies from Pubmed and Embase database up to January 10, 2018. ORs and 95% CIs were used to estimate the associations between miR-499 polymorphism and cancer susceptibility in different systems. All analyses were performed using the Stata 11.0 software. A total of 65 case-control studies were retrieved using explicit inclusion and exclusion criteria. The study included 23,762 cases and 28,694 controls. Overall cancer analysis showed the association between miR-499 polymorphism and susceptibility to cancer was significant. MicroRNA-499 rs3746444 was found to be significantly associated with increased risk of cancer of the respiratory system (CC vs. TT: OR = 1.575, 95% CI = 1.268–1.955, CC vs. TC+TT: OR = 1.527, 95% CI = 1.232–1.892), digestive system (CC vs. TT: OR = 1.153, 95% CI = 1.027–1.295; TC vs. TT: OR = 1.109, 95% CI = 1.046–1.176; CC+TC vs. TT: OR = 1.112, 95% CI = 1.018–1.216; CC vs. TC+TT: OR = 1.137, 95% CI = 1.016–1.272; C vs. T: OR = 1.112, 95% CI = 1.025–1.206), urinary system (TC vs. TT: OR = 1.307, 95% CI = 1.130–1.512; CC+TC vs. TT: OR = 1.259, 95% CI = 1.097–1.446; C vs. T: OR = 1.132, 95% CI = 1.014–1.264), and gynecological system (C vs. T: OR = 1.169, 95% CI = 1.002–1.364). In the subgroup analysis by ethnicity, the result showed that significant association with an increased cancer risk was found in Asian. Subgroup analysis based on type of tumor was also performed, miR-499 rs3746444 is associated with susceptibility of cervical squamous cell carcinoma, lung cancer, prostate cancer, and hepatocellular carcinoma.

## Introduction

Cancer is a chronic disease that severely affects the lives and health of people in most countries and regions worldwide. The World Health Organization's International Agency for Research on Cancer recently published cancer statistics indicating that: 14.1 million new cancer cases, 8.4 million deaths occurred in 2012 worldwide (Torre et al., 2015). There is a growing body of evidence showing that carcinogenic effects are complex processes involving multiple environmental and genetic factors, although the cause of carcinogenic effects has not yet been completely clarified.

Micro-ribonucleic acids (miRNAs) are ~19- to 24-nucleotide RNA molecules that can regulate the expression of other genes in eukaryotes (Bartel, 2004). A growing number of studies have shown that miRNAs play an important role in regulating a variety of cellular functions. Impairment of miRNA expression is usually associated with various diseases. MicroRNAs play an important regulatory role in tumorigenesis through the regulation of cancer-related target gene expression and pathways. A variety of evidence suggests that miRNAs can participate in immune responses; inflammatory responses; infections; and cellular metabolism, growth and migration (Jovanovic and Hengartner, 2006).

MiR-499 is one of the microRNAs that plays important posttranscriptional regulatory roles by modulating several genes and signaling pathways, particularly under hypoxic-ischemic conditions such as in cancer and myocardial infarction (Wilson et al., 2010; Ando et al., 2014). Liu et al. (2011) indicated that miR-499 could promote metastasis of colorectal cancer cells by targeting FOXO4 and PDCD4, and miR-499 might be regarded as a new potential therapeutic target for colorectal cancer. Li et al. also found that miR-499 functions as a tumor suppressor in non-small cell lung cancer by targeting VAV3 (Li M. et al., 2016). MiR-499 is related to several signaling pathways such as wnt/β-catenin signal pathway (Zhang et al., 2012). A study by Wang et al. (2015) suggested that downregulation of the miR-499-5p level impaired the PI3K/AKT/GSK signaling pathway and glycogen synthesis by targeting PTEN. Ando et al. (2014) reported that the tumor-targeted delivery of miR-499 was very effective so as to implement cancer therapy. Okamoto et al. (2016) found that APRPG-miR-499 may be a combination therapeutic agent for cancer treatment.

Single nucleotide genetic variants that occur in miRNAs may influence microRNA biogenesis, stability of mature microRNA molecules, efficiency of target gene regulation, as well as specificity of targets, which may involve developing susceptibility to cancer, thus, miRNAs play an important role in the occurrence and development of malignant tumors (Nikolic et al., 2015).

Accumulated evidence suggests that miR-499 rs3746444 is associated with the susceptibility to many cancers. Zhang et al. (2017) reported that the miR-499 rs3746444 polymorphism is associated with the risk of oral squamous cell cancer in Chinese individuals. Hashemi et al. (2016) found that the rs3746444 polymorphism in miR-499 can increase the risk of prostate cancer in an Iranian population. The rs3746444 single nucleotide polymorphism was also found to be clearly associated with lung cancer (Li D. et al., 2016). Wang et al. (2014) found that miR-499A>G polymorphism is associated with an increased risk of hepatocellular carcinoma in Chinese individuals. However, Zhang et al. (2016) suggested that there is no association between miR-499 variant and the risk of hepatocellular carcinoma in the co-dominant, dominant, and recessive models. These two articles similarly focus on hepatocellular carcinoma, but it led to very different results. In a Greek population, a case–control study demonstrated that the rs3746444 polymorphism in miR-499 is not associated with colorectal cancer (Dikaiakos et al., 2015). For this gene locus, many investigators have conducted exploratory studies and also obtained a number of different results, different outcomes for different types of cancer, and even the same cancer may have different results. The association between miR-499 rs3746444 and the susceptibility to cancer has not yet reached a clear consensus.

Now that increasingly more and more researchers have studied the association between cancer risk and miR-499 polymorphism, it is necessary to integrate these results to further explore the relationship between the miR-499 polymorphism and cancer risk of different systems at the level of statistics.

## Materials and methods

### Literature retrieval

We retrieved articles from PubMed and EMBASE using the following terms “microRNA-499 or miR-499 or rs3746444” and “cancer or carcinoma” published prior to January 10, 2018. In order to be more comprehensive and rigorous, we searched for the documents mentioned in the original article and the references to find more qualified article. There is no language restriction on the retrieved literature.

### Study selection and data extraction

All incorporated literature must clearly meet the following three criteria: (a) evaluation of the miR-499 rs3746444 polymorphism and cancer risks; (b) a case-control study; and (c) sufficient published data for the computation of odds ratios (ORs) with 95% confidence intervals (CIs). The exclusion criteria were as follows: (a) Animal studies; (b) not for cancer research; (c) meta-analyses or review articles; Two reviewers independently extract data. If the opinion is inconsistent, the third review will join the in-depth discussion to determine whether the article should be obtained. The main contents of extracted information included the name of first author, publication date, country of origin, ethnicity, cancer type, characteristics of controls, the number of cases and controls with miR-499 T/C genotypes and the total number of cases and controls. According to the source of the control, we defined controls as population-based and hospital-based.

### Statistical methods

We firstly calculated the value of Hardy-Weinberg equilibrium (HWE) of the control groups by using the chi-square goodness-of-fit test including all retrieved literature (a *P* < 0.05 was regarded representative of a departure from HWE). ORs corresponding to 95% CI was used to estimate the strength of association between miR-499 rs3746444 T/C and different cancer risk. The pooled ORs were conducted for five genetic comparison models: allelic model (C vs. T), homozygote comparison (CC vs. TT), heterozygote comparison (TC vs. TT), recessive model (CC vs. TC/TT), dominant model (CC/TC vs. TT). Heterogeneity among retrieved literature was used to evaluate by *I*^2^ test and Q test (a significance level of *P* < 0.05 and/or *I*^2^ ≥ 50%). If there was obvious heterogeneity (*I*^2^ ≥ 50%), we conducted with a random-effects model (DerSimonian and Laird), otherwise, we analyzed with a fixed-effects model (Mantel–Haenszel) to obtain summary associations between miR-499 rs3746444 T/C and different cancer risk. Then subgroup analysis was done by ethnicity, different systems, source of control, and type of tumor, HWE in controls (yes/no). Sensitivity analyses were used to evaluate the stabilization of the results. Potential effect of publication bias of literatures was estimated by funnel plots and the Egger's test. A *P* < 0.05 or the unsymmetrical funnel plot were regarded as statistically significant. Statistical analysis was done by Stata11.0 Software. All the *P*-values are two-sided.

## Results

### Study characteristics

**Figure 1:**
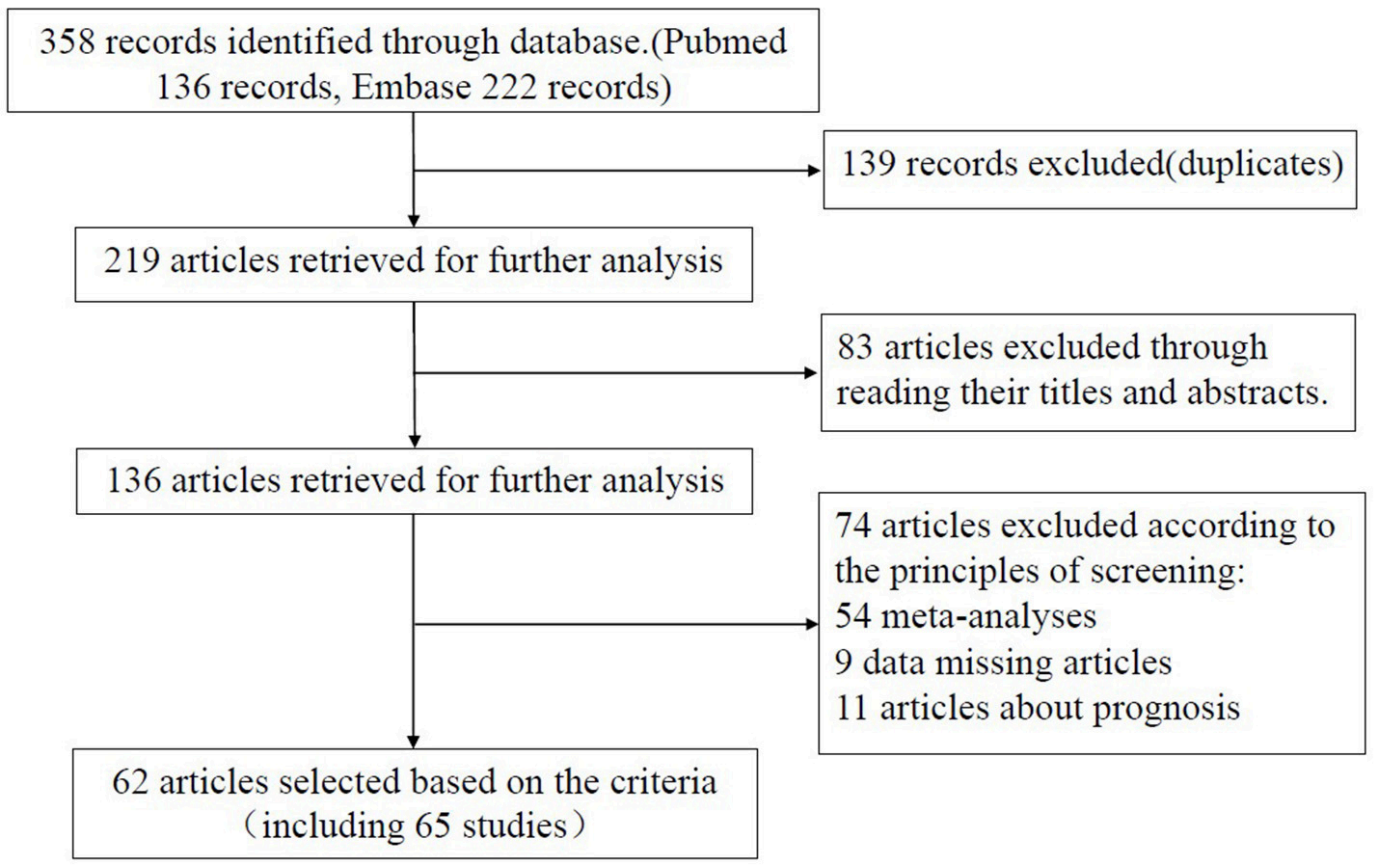
Flow chart of the study selection process.

We first obtained 219 articles by using our specified search terms (Figure 1). After reading the titles and the abstracts, 83 articles were excluded. To meet the screening criteria set out earlier, we also excluded an additional 74 articles, including 54 meta-analyses, 9 articles with missing data, and 11 articles concerning prognosis. Finally, a total of 62 articles (65 studies) consisting of 23,762 cases and 28,694 controls were used for further analysis. Of the 65 studies, sample sizes ranged from 175 to 3,585, and publication year ranged from 2009 to 2017. In total, there were 2 acute lymphoblastic leukemia studies (Gutierrez-Camino et al., 2014; Hasani et al., 2014), 10 breast cancer studies (Hu et al., 2009; Catucci et al., 2010; Alshatwi et al., 2012; Bansal et al., 2014; Omrani et al., 2014; He et al., 2015; Qi et al., 2015; Dai et al., 2016; Afsharzadeh et al., 2017), 3 bladder cancer studies (Mittal et al., 2011; Deng et al., 2015; Wang et al., 2016), 5 colorectal cancer studies (Min et al., 2012; Vinci et al., 2013; Hu et al., 2014; Dikaiakos et al., 2015; Ying et al., 2016), 2 cervical squamous cell carcinoma studies (Zhou et al., 2011; Srivastava et al., 2017), 3 esophageal cancer studies (Umar et al., 2013; Wei et al., 2013; Shen et al., 2016), 7 gastric cancer studies (Okubo et al., 2010; Ahn et al., 2013; Wu et al., 2013; Pu et al., 2014; Cai et al., 2015; Poltronieri-Oliveira et al., 2017; Rogoveanu et al., 2017), 17 hepatocellular carcinoma studies (Akkiz et al., 2011; Kim et al., 2012; Xiang et al., 2012; Zhou et al., 2012, 2014; Shan et al., 2013; Zou and Zhao, 2013; Chu et al., 2014; Hao et al., 2014; Kou et al., 2014; Ma et al., 2014; Qi et al., 2014; Wang et al., 2014; Li D. et al., 2015; Li X. et al., 2015; Yan et al., 2015; Zhang et al., 2016), 4 lung cancer studies (Tian et al., 2009; Vinci et al., 2011; Li D. et al., 2016), 2 oral squamous cell cancer studies (Hou et al., 2015; Zhang et al., 2017), 3 prostate cancer (George et al., 2011; Nikolic et al., 2015; Hashemi et al., 2016), and other cancer studies [1 cervical squamous cell carcinoma study (Zhou et al., 2011), 1 endometrial cancer study (Liu et al., 2015), 1 gallbladder cancer study (Srivastava et al., 2010), 1 ovarian cancer study (Liu et al., 2015), 1 nasopharyngeal carcinoma study (Qiu et al., 2015), 1 renal cell cancer study (Du et al., 2014), 1 cell carcinoma of head and neck study (Liu et al., 2010)]. There were 22 studies on Caucasians and 43 studies on Asians. Table 1 indicates the main characteristics of each study included in this meta-analysis. We separated these studies into several categories, such as cancers of the respiratory system (8 studies), digestive system (33 studies), urinary system (7 studies), gynecological system (14 studies), and lymphatic and hematopoietic system (2 studies).

**Table 1:** Characteristics of all the studies in the meta-analysis.

**Authors**	**Year**	**Country**	**Ethnicity**	**Cancer type**	**Study design**	**Number cases/controls**	**Cases**			**Controls**			
							**TT**	**TC**	**CC**	**TT**	**TC**	**CC**	**HWE**
Zhang	2017	China	Asian	OSCC	HB	340/340	191	118	31	217	111	12	0.633
Srivastava	2017	India	Caucasian	CSCC	PB	184/164	26	78	80	54	76	34	0.448
Rogoveanu	2017	Romania	Caucasian	GC	HB	142/288	80	58	4	173	107	8	0.072
Poltronieri-Oliveira	2017	Brazil	Caucasian	GC	HB	150/239	97	48	5	143	90	6	0.060
Afsharzadeh	2017	Iran	Caucasian	BC	HB	100/150	63	33	4	66	65	19	0.633
Hashemi	2016	Iran	Caucasian	PC	HB	169/182	62	82	25	85	64	33	0.002
Li	2016	China	Asian	LC	PB	500/500	316	149	35	350	130	20	0.079
Li	2016	China	Asian	LC	PB	700/700	461	195	44	500	172	28	0.009
Zhang	2016	China	Asian	HCC	HB	175/302	115	49	11	197	87	18	0.052
Shen	2016	China	Asian	ESCC	PB	1400/2185	1019	352	29	1646	492	47	0.155
Ying	2016	China	Asian	CRC	PB	1350/1075	872	336	142	713	245	117	< 0.001
Wang	2016	China	Asian	BLC	PB	283/283	190	70	23	203	70	10	0.206
Dai	2016	China	Asian	BC	HB	560/583	407	135	18	463	109	11	0.131
Nikoli	2015	Serbia	Caucasian	PC	PB	355/307	190	147	18	180	110	17	0.971
Cai	2015	China	Asian	GC	PB	363/969	261	89	13	765	179	25	< 0.001
Li	2015	China	Asian	HCC	PB	266/250	150	92	24	166	83	17	0.14
Liu	2015	China	Asian	EC	PB	141/100	123	18	0	77	23	0	0.194
Liu	2015	China	Asian	OC	PB	75/100	58	17	0	77	23	0	0.194
Qi	2015	China	Asian	BC	HB	321/290	152	117	52	141	112	37	0.053
He	2015	China	Asian	BC	HB	450/450	184	177	89	203	188	59	0.143
Deng	2015	China	Asian	BLC	PB	159/298	107	45	7	216	68	14	0.007
Dikaiakos	2015	Greece	Caucasian	CRC	HB	157/299	85	64	8	182	99	18	0.361
Li	2015	China	Asian	HCC	HB	184/184	117	43	24	128	39	17	< 0.001
Qiu	2015	China	Asian	NPC	PB	906/1072	614	243	49	750	284	38	0.089
Yan	2015	China	Asian	HCC	PB	274/328	147	98	29	188	112	28	0.06
Hou	2015	China	Asian	OSCC	HB	512/668	394	109	9	464	192	12	0.119
Wang	2014	China	Asian	HCC	HB	152/304	98	32	22	218	62	24	< 0.001
Bansal	2014	India	Caucasian	BC	PB	121/164	80	30	11	106	43	15	0.002
Omrani	2014	Iran	Caucasian	BC	PB	236/203	131	44	61	130	48	25	< 0.001
Du	2014	China	Asian	RCC	HB	354/362	251	94	9	255	96	11	0.594
Hasani	2014	Iran	Caucasian	ALL	HB	75/115	35	28	12	61	42	12	0.249
Qi	2014	China	Asian	HCC	PB	314/406	195	117	2	301	101	4	0.157
Ma	2014	China	Asian	HCC	PB	984/991	724	241	19	765	179	25	< 0.001
Chu	2014	China	Asian	HCC	HB	188/337	119	60	9	281	55	1	0.321
Gutierrezcamino	2014	Spain	Caucasian	ALL	PB	200/347	138	56	6	206	117	24	0.194
Hu	2014	China	Asian	CRC	HB	211/373	157	49	5	282	81	10	0.162
Kou	2014	China	Asian	HCC	HB	271/532	210	49	12	391	110	31	< 0.001
Hao	2014	China	Asian	HCC	HB	235/281	160	51	24	204	61	16	< 0.001
Zhou	2014	China	Asian	HCC	HB	266/281	184	59	23	204	61	16	< 0.001
Zou	2013	China	Asian	HCC	HB	185/185	136	44	5	123	48	14	0.005
Wu	2013	China	Asian	GC	HB	200/211	149	47	4	166	42	3	0.854
Wei	2013	China	Asian	ESCC	HB	358/376	291	60	7	289	76	11	0.036
Vinci	2013	Italy	Caucasian	CRC	HB	160/178	93	32	35	105	56	17	0.026
Shan	2013	China	Asian	HCC	HB	172/185	128	37	7	123	48	14	0.005
Umar	2013	India	Caucasian	ESCC	PB	289/309	155	122	12	149	140	20	0.087
Pu	2013	China	Asian	GC	HB	196/504	141	50	5	366	121	17	0.082
Ahn	2013	Korea	Asian	GC	HB	461/447	323	123	15	299	134	14	0.829
Zhou	2012	China	Asian	HCC	PB	186/483	141	41	4	371	100	12	0.1
Xiang	2012	China	Asian	HCC	HB	100/100	36	40	24	52	35	13	0.081
Min	2012	Korea	Asian	CRC	PB	446/502	292	142	12	334	154	14	0.453
Kim	2012	Korea	Asian	HCC	PB	159/201	109	47	3	120	74	7	0.278
Chu	2012	China	Asian	OSCC	HB	470/425	339	119	12	356	66	3	0.975
Alshatwi	2012	Saudi Arabia	Caucasian	BC	HB	100/100	30	62	8	45	40	15	0.227
Zhou	2011	China	Asian	CSCC	PB	226/309	134	84	8	223	71	15	0.005
Vinci	2011	Italy	Caucasian	LC	HB	101/129	53	41	7	70	48	11	0.503
Mittal	2011	India	Caucasian	BLC	PB	212/250	95	92	25	121	94	35	0.02
Akkiz	2011	Turkey	Caucasian	HCC	PB	222/222	45	87	90	47	93	82	0.036
George	2011	India	Caucasian	PC	PB	159/230	48	98	13	104	92	34	0.073
Srivastava	2010	India	Caucasian	GBC	PB	230/230	112	97	21	121	94	15	0.566
Okubo	2010	Japan	Asian	GC	HB	552/697	364	151	37	466	198	33	0.048
Catucci	2010	Italy	Caucasian	BC	PB	756/1242	414	295	47	704	452	86	0.25
Catucci	2010	Germany	Caucasian	BC	PB	823/925	536	250	37	601	290	34	0.893
Liu	2010	USA	Caucasian	SCCHN	HB	1109/1130	745	309	55	710	366	54	0.441
Tian	2009	China	Asian	LC	PB	1058/1035	781	253	24	755	254	26	0.404
Hu	2009	China	Asian	BC	PB	1009/1093	707	258	44	816	248	29	0.057
Chu	2014	China	Asian	HCC	HB	188/337	119	60	9	281	55	1	0.321

*ALL, acute lymphoblastic leukemia; BC, breast cancer; BLC, bladder cancer; CRC, colorectal cancer; CSCC, cervical squamous cell carcinoma; EC, endometrial cancer; ESCC, esophageal cancer; GC, gastric cancer; GBC, gallbladder cancer; HCC, hepatocellular carcinoma; LC, lung cancer; NPC, nasopharyngeal carcinoma; OC, ovarian cancer; OSCC, oral squamous cell cancer; PC, prostate cancer; RCC, renal cell cancer; SCCHN, cell carcinoma of head and neck*.

### Meta-analysis results

As is shown in Table 2, the main analyses performed included association and heterogeneity tests. Among all studies, we found significant associations between miR-499 rs3746444 polymorphism and cancer susceptibility in five kinds of genetic models. (CC vs. TT: OR = 1.227, 95% CI = 1.084–1.388, *P* = 0.001; TC vs. TT: OR = 1.108, 95% CI = 1.035–1.186, *P* = 0.003; CC+TC vs. TT: OR = 1.136, 95% CI = 1.061–1.216, *P* < 0.001; CC vs. TC+TT: OR = 1.177, 95% CI = 1.045–1.326, *P* = 0.007; C vs. T: OR = 1.126, 95% CI = 1.059–1.197, *P* < 0.001).

**Table 2:** Pooled ORs and 95% CIs for microRNA-499 polymorphism of stratified meta-analysis.

**Subgroup**	**Genotype**	**No**.	**Test of association**				**Test of heterogeneity**		
			**OR (95%CI)**	**Z**	***P*-value**	**Model**	**χ2**	***P*-value**	***I*^2^ (%)**
TOTAL	CC vs. TT	65	1.227 (1.084, 1.388)	3.25	**0.001**	R	126.21	< 0.001	50.9
	TC vs. TT	65	1.108 (1.035, 1.186)	2.97	**0.003**	R	163.27	< 0.001	60.8
	CC+TC vs. TT	65	1.136 (1.061, 1.216)	3.66	<**0.001**	R	186.38	< 0.001	65.7
	CC vs. TC+TT	65	1.177 (1.045, 1.326)	2.68	**0.007**	R	124.88	< 0.001	50.4
	C vs. T	65	1.126 (1.059, 1.197)	3.82	<**0.001**	R	217.32	< 0.001	70.6
Respiratory system	CC vs. TT	8	1.575 (1.268, 1.955)	4.11	<**0.001**	F	13.03	0.071	46.3
	TC vs. TT	8	1.118 (0.923, 1.354)	1.14	0.253	R	27.04	< 0.001	74.1
	CC+TC vs. TT	8	1.171 (0.956, 1.435)	1.52	0.128	R	33.52	< 0.001	79.1
	CC vs. TC+TT	8	1.527 (1.232, 1.892)	3.86	<**0.001**	F	11.10	0.134	37.0
	C vs. T	8	1.188 (0.987, 1.429)	1.82	0.069	R	37.55	< 0.001	81.4
Digestive system	CC vs. TT	33	1.153 (1.027,1.295)	2.41	**0.016**	F	48.54	0.031	34.1
	TC vs. TT	33	1.109 (1.046,1.176)	3.47	**0.001**	F	63.88	0.001	49.9
	CC+TC vs. TT	33	1.112 (1.018,1.216)	2.34	**0.019**	R	74.47	< 0.001	57.0
	CC vs. TC+TT	33	1.137 (1.016,1.272)	2.24	**0.025**	R	46.30	0.049	30.9
	C vs. T	33	1.112 (1.025,1.206)	2.55	**0.011**	R	89.63	< 0.001	64.3
Urinary system	CC vs. TT	7	1.068 (0.818,1.394)	0.49	0.627	F	5.64	0.465	0.0
	TC vs. TT	7	1.307 (1.130,1.512)	3.61	<**0.001**	F	9.57	0.072	48.1
	CC+TC vs. TT	7	1.259 (1.097,1.446)	3.27	**0.001**	F	6.97	0.324	13.9
	CC vs. TC+TT	7	0.888 (0.689,1.144)	0.92	0.358	F	9.47	0.149	36.6
	C vs. T	7	1.132 (1.014,1.264)	2.21	**0.027**	F	3.33	0.767	0.0
Gynecological system	CC vs. TT	14	1.369 (0.990, 1.892)	1.90	0.057	R	40.28	< 0.001	72.7
	TC vs. TT	14	1.122 (0.951, 1.324)	1.36	0.174	R	39.02	< 0.001	66.7
	CC+TC vs. TT	14	1.180 (0.995, 1.400)	1.91	0.057	R	47.41	< 0.001	72.6
	CC vs. TC+TT	14	1.279 (0.950, 1.724)	1.62	0.105	R	37.62	< 0.001	70.8
	C vs. T	14	1.169 (1.002, 1.364)	1.99	**0.047**	R	60.95	< 0.001	78.7
Lymphatic and hematopoietic system	CC vs. TT	2	0.809 (0.179,3.662)	0.28	0.783	R	5.50	0.019	81.8
	TC vs. TT	2	0.813 (0.586,1.128)	1.24	0.216	F	1.65	0.198	39.5
	CC+TC vs. TT	2	0.885 (0.458,1.710)	0.36	0.716	R	3.70	0.055	72.9
	CC vs. TC+TT	2	0.832 (0.218,3.180)	0.27	0.788	R	4.58	0.032	78.2
	C vs. T	2	0.914 (0.462,1.808)	0.26	0.796	R	6.36	0.012	84.3
Caucasian	CC vs. TT	22	1.110 (0.874,1.410)	0.85	0.394	R	57.01	< 0.001	63.2
	TC vs. TT	22	1.082(0.942,1.242)	1.12	0.263	R	60.00	< 0.001	65.0
	CC+TC vs. TT	22	1.106 (0.968,1.263)	1.48	0.139	R	63.40	< 0.001	66.9
	CC vs. TC+TT	22	1.038 (0.823,1.310)	0.32	0.751	R	60.65	< 0.001	65.4
	C vs. T	22	1.077 (0.963,1.205)	1.30	0.195	R	75.17	< 0.001	72.1
Asian	CC vs. TT	43	1.301 (1.180, 1.434)	5.28	<**0.001**	F	67.39	0.004	40.6
	TC vs. TT	43	1.123 (1.040, 1.213)	2.97	**0.003**	R	99.45	< 0.001	57.8
	CC+TC vs. TT	43	1.152 (1.065, 1.247)	3.52	<**0.001**	R	118.06	< 0.001	64.4
	CC vs. TC+TT	43	1.267 (1.151, 1.394)	4.83	<**0.001**	F	61.94	0.015	35.4
	C vs. T	43	1.151 (1.072, 1.237)	3.85	<**0.001**	R	135.59	< 0.001	69.0
PB	CC vs. TT	32	1.216 (1.032,1.434)	2.33	**0.020**	R	62.80	< 0.001	53.8
	TC vs. TT	32	1.152 (1.063,1.249)	3.45	**0.001**	R	66.88	< 0.001	53.7
	CC+TC vs. TT	32	1.169 (1.079,1.267)	3.81	<**0.001**	R	74.54	< 0.001	58.4
	CC vs. TC+TT	32	1.151 (0.983,1.348)	1.74	0.081	R	62.46	< 0.001	53.6
	C vs. T	32	1.146 (1.066,1.231)	3.69	<**0.001**	R	88.23	< 0.001	64.9
HB	CC vs. TT	33	1.283 (1.135, 1.450)	3.98	<**0.001**	F	63.03	0.001	49.2
	TC vs. TT	33	1.065 (0.952, 1.192)	1.11	0.269	R	89.38	< 0.001	64.2
	CC+TC vs. TT	33	1.104 (0.984, 1.239)	1.68	0.093	R	107.48	< 0.001	70.2
	CC vs. TC+TT	33	1.262 (1.120, 1.422)	3.82	<**0.001**	F	61.52	0.001	48.0
	C vs. T	33	1.108 (0.999, 1.229)	1.93	0.053	R	127.45	< 0.001	74.9
HWE YES	CC vs. TT	44	1.241 (1.054,1.461)	2.59	**0.010**	R	87.07	< 0.001	52.9
	TC vs. TT	44	1.109 (1.019,1.208)	2.39	**0.017**	R	123.62	< 0.001	65.2
	CC+TC vs. TT	44	1.133 (1.037,1.238)	2.76	**0.006**	R	148.27	< 0.001	71.0
	CC vs. TC+TT	44	1.230 (1.115,1.356)	4.14	<**0.001**	F	80.13	< 0.001	48.8
	C vs. T	44	1.120 (1.036,1.211)	2.84	**0.004**	R	166.32	< 0.001	74.1
HWE NO	CC vs. TT	21	1.203 (1.203,1.364)	2.89	**0.004**	F	38.85	0.007	48.5
	TC vs. TT	21	1.139 (1.056,1.229)	3.38	**0.001**	F	38.35	0.008	47.9
	CC+TC vs. TT	21	1.158 (1.080,1.241)	4.14	<**0.001**	F	36.77	0.012	45.6
	CC vs. TC+TT	21	1.163 (0.956,1.415)	1.51	0.132	R	44.40	0.001	55.0
	C vs. T	21	1.141 (1.037,1.254)	2.72	**0.007**	R	50.05	< 0.001	60.0
ALL	CC vs. TT	2	0.809 (0.179,3.662)	0.28	0.783	R	5.50	0.019	81.8
	TC vs. TT	2	0.813 (0.586,1.128)	1.24	0.216	F	1.65	0.198	39.5
	CC+TC vs. TT	2	0.885 (0.458,1.710)	0.36	0.716	R	3.70	0.055	72.9
	CC vs. TC+TT	2	0.832 (0.218,3.180)	0.27	0.788	R	4.58	0.032	78.2
	C vs. T	2	0.914 (0.462,1.808)	0.26	0.796	R	6.36	0.012	84.3
BC	CC vs. TT	10	1.275 (0.956,1.700)	1.65	0.098	R	23.22	0.006	61.2
	TC vs. TT	10	1.077 (0.930,1.246)	0.99	0.322	R	19.44	0.022	53.7
	CC+TC vs. TT	10	1.130 (0.974,1.311)	1.61	0.107	R	23.17	0.006	61.1
	CC vs. TC+TT	10	1.230 (0.918,1.646)	1.39	0.165	R	25.37	0.033	64.5
	C vs. T	10	1.132 (0.982,1.304)	1.71	0.087	R	32.71	< 0.001	72.5
BLC	CC vs. TT	3	1.292 (0.684,2.442)	0.79	0.429	R	4.33	0.115	53.8
	TC vs. TT	3	1.200 (0.950,1.517)	1.53	0.126	F	0.61	0.737	0.0
	CC+TC vs. TT	3	1.220 (0.981,1.519)	1.79	0.074	F	0.15	0.930	0.0
	CC vs. TC+TT	3	1.208 (0.607,2.406)	0.54	0.591	R	5.28	0.071	62.1
	C vs. T	3	1.174 (0.984,1.401)	1.78	0.075	F	1.78	0.410	0.0
CRC	CC vs. TT	5	1.094 (0.879,1.362)	0.81	0.420	F	6.10	0.192	34.4
	TC vs. TT	5	1.085 (0.950,1.240)	1.20	0.231	F	5.43	0.246	26.3
	CC+TC vs. TT	5	1.089 (0.964,1.230)	1.38	0.169	F	1.06	0.900	0.0
	CC vs. TC+TT	5	1.161 (0.747,1.805)	0.66	0.506	R	9.25	0.055	56.8
	C vs. T	5	1.078 (0.975,1.191)	1.47	0.143	F	2.84	0.586	0.0
CSCC	CC vs. TT	2	2.148 (0.404,11.415)	0.90	0.370	R	9.62	0.002	89.6
	TC vs. TT	2	2.070 (1.472,2.771)	4.36	<**0.001**	F	0.05	0.819	0.0
	CC+TC vs. TT	2	2.221 (1.346,3.665)	3.12	**0.002**	R	2.49	0.114	59.9
	CC vs. TC+TT	2	1.529 (0.386,6.057)	0.60	0.546	R	7.66	0.006	86.9
	C vs. T	2	1.845 (1.158,2.940)	2.58	**0.010**	R	4.62	0.032	78.3
ESCC	CC vs. TT	3	0.815 (0.564,1.177)	1.09	0.275	F	1.79	0.408	0.0
	TC vs. TT	3	0.950 (0.725,1.245)	0.37	0.712	R	5.53	0.063	63.8
	CC+TC vs. TT	3	0.921 (0.691,1.226)	0.57	0.572	R	6.66	0.036	70.0
	CC vs. TC+TT	3	0.818 (0.568,1.178)	1.08	0.280	F	1.16	0.560	0.0
	C vs. T	3	0.915 (0.712,1.174)	0.70	0.483	R	6.99	0.030	71.4
GC	CC vs. TT	7	1.251 (0.925,1.692)	1.45	0.146	F	2.01	0.919	0.0
	TC vs. TT	7	1.052 (0.926,1.194)	0.77	0.439	F	9.73	0.136	38.4
	CC+TC vs. TT	7	1.074 (0.951,1.214)	1.16	0.248	F	9.59	0.143	37.5
	CC vs. TC+TT	7	1.243 (0.922,1.676)	1.43	0.154	F	1.83	0.935	0.0
	C vs. T	7	1.083 (0.975,1.203)	1.49	0.135	F	8.63	0.195	30.5
HCC	CC vs. TT	17	1.197 (0.902,1.579)	1.28	0.202	R	33.65	0.006	52.5
	TC vs. TT	17	1.146 (0.981,1.339)	1.72	0.085	R	39.67	0.001	59.7
	CC+TC vs. TT	17	1.178 (0.998,1.390)	1.94	0.053	R	52.18	< 0.001	69.3
	CC vs. TC+TT	17	1.206 (1.022,1.423)	2.21	**0.027**	F	29.51	0.021	45.8
	C vs. T	17	1.169 (1.005,1.360)	2.02	**0.043**	R	64.61	< 0.001	75.2
LC	CC vs. TT	4	1.392 (1.039, 1.866)	2.21	**0.027**	F	5.29	0.152	43.3
	TC vs. TT	4	1.110 (0.974, 1.266)	1.56	0.118	F	3.50	0.320	14.4
	CC+TC vs. TT	4	1.161 (0.959, 1.406)	1.53	0.126	R	6.13	0.105	51.1
	CC vs. TC+TT	4	1.335 (0.999, 1.785)	1.95	0.051	F	4.60	0.203	34.8
	C vs. T	4	1.157 (0.951, 1.407)	1.46	0.145	R	8.79	0.032	65.9
OSCC	CC vs. TT	3	2.116 (0.857,5.229)	1.62	0.104	R	5.80	0.055	65.5
	TC vs. TT	3	1.145 (0.623,2.103)	0.44	0.663	R	23.24	< 0.001	91.4
	CC+TC vs. TT	3	1.275 (0.648,2.316)	0.62	0.533	R	27.31	< 0.001	92.7
	CC vs. TC+TT	3	2.051 (0.951,4.424)	1.83	0.067	R	4.28	0.118	53.2
	C vs. T	3	1.267 (0.715,2.247)	0.81	0.417	R	28.63	< 0.001	93.0
PC	CC vs. TT	3	0.962 (0.653,1.417)	0.20	0.844	F	0.24	0.088	0.0
	TC vs. TT	3	1.678 (1.167,2.414)	2.79	**0.005**	R	4.81	0.090	58.4
	CC+TC vs. TT	3	1.453 (1.172,1.802)	3.40	**0.001**	F	2.72	0.257	26.4
	CC vs. TC+TT	3	0.717 (0.499,1.030)	1.80	0.072	F	1.51	0.469	0.0
	C vs. T	3	1.158 (0.985,1.362)	1.77	0.076	F	0.08	0.961	0.0
Other	CC vs. TT	6	1.203 (0.931,1.554)	1.41	0.158	F	3.68	0.298	18.4
	TC vs. TT	6	0.919 (0.818,1.032)	1.43	0.151	F	8.11	0.150	38.3
	CC+TC vs. TT	6	0.950 (0.851,1.061)	0.90	0.367	F	9.90	0.078	49.5
	CC vs. TC+TT	6	1.227 (0.952,1.581)	1.58	0.113	F	2.80	0.424	0.0
	C vs. T	6	0.986 (0.837,1.161)	0.17	0.862	R	10.87	0.054	54.0

*OR, odds ratio; vs., versus; CI, confidence interval; P, p-value of Z-test for pooled OR; P_h_, p-value of heterogeneity test; PB, population-based; HB, hospital-based; F, fixed effect model; R, random effect model; ALL, acute lymphoblastic leukemia; BC, breast cancer; BLC, bladder cancer; CRC, colorectal cancer; CSCC, cervical squamous cell carcinoma; EC, endometrial cancer; ESCC, esophageal cancer; GC, gastric cancer; GBC, gallbladder cancer; HCC, hepatocellular carcinoma; LC, lung cancer; NPC, nasopharyngeal carcinoma; OC, ovarian cancer; OSCC, oral squamous cell cancer; PC, prostate cancer; RCC, renal cell cancer; SCCHN, cell carcinoma of head and neck. Bold values are significant to P < 0.05*.

**Figure 2:**
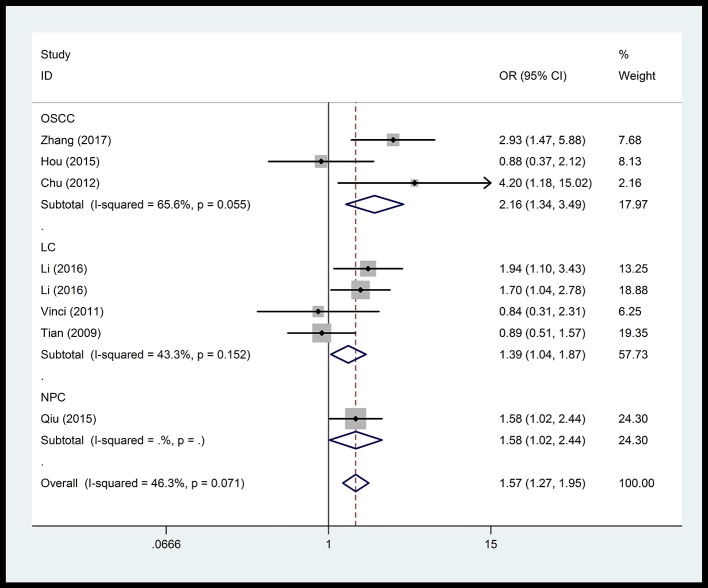
Forest plot of the OR and 95% CIs for association between microRNA-499 rs3746444 polymorphism and cancer risk of respiratory system in homozygote comparison model. OSCC, oral squamous cell cancer; LC, lung cancer; NPC, nasopharyngeal carcinoma.

**Figure 3:**
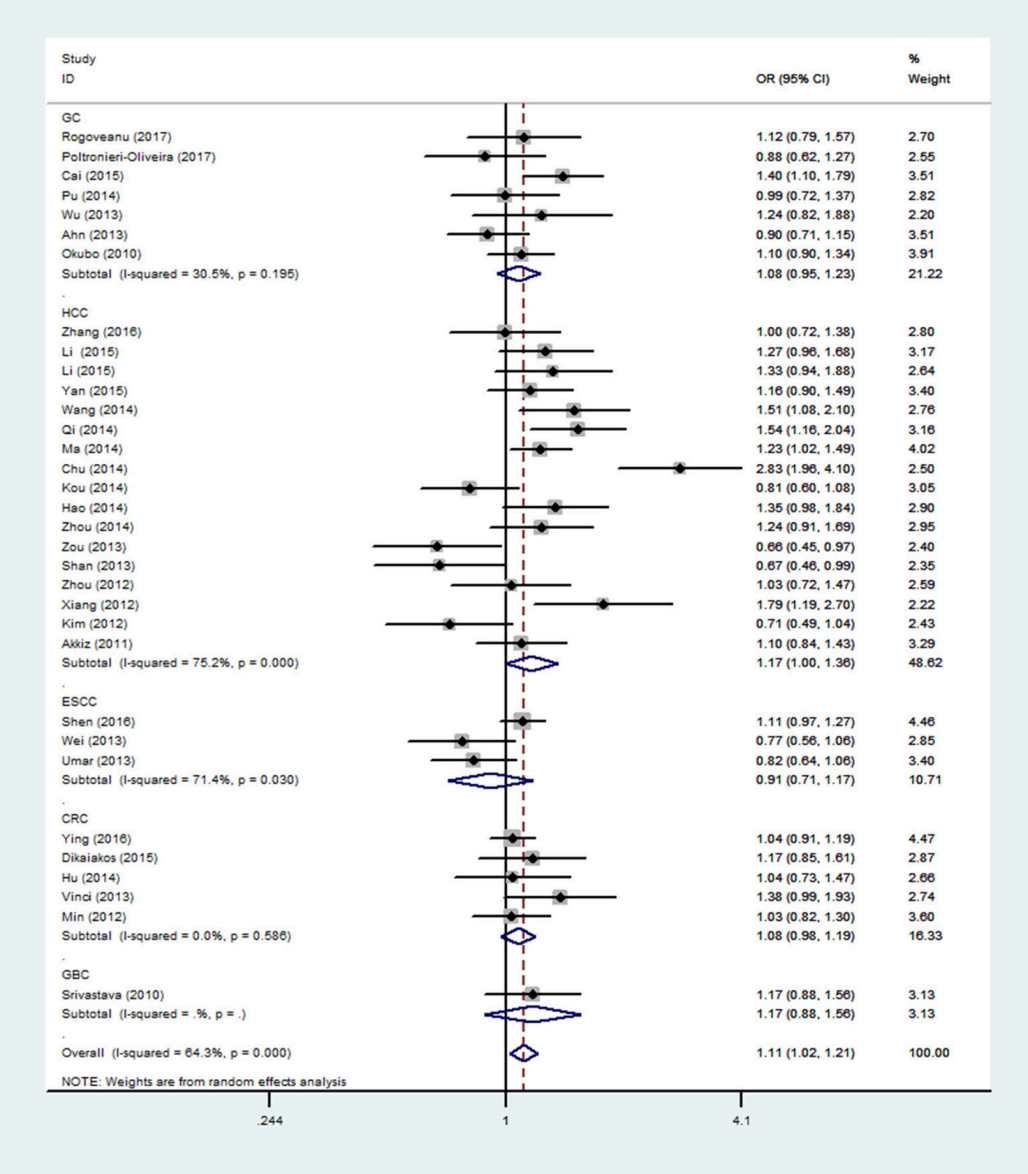
Forest plot of the OR and 95% CIs for association between microRNA-499 rs3746444 polymorphism and cancer risk of digestive system in allelic model. HCC, hepatocellular carcinoma; ESCC, esophageal cancer; CRC, colorectal cancer; GC, gastric cancer; GBC, gallbladder cancer.

**Figure 4:**
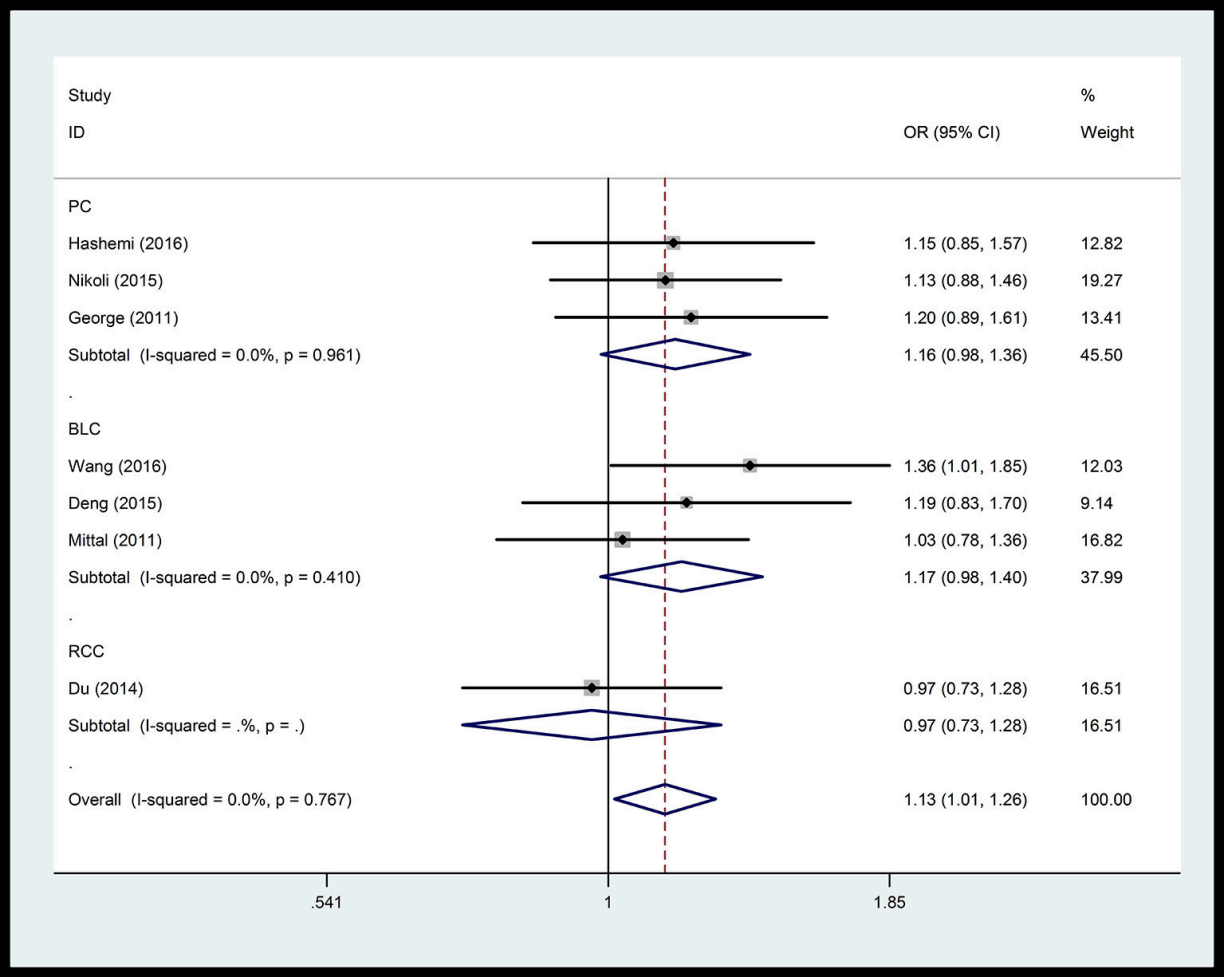
Forest plot of the OR and 95% CIs for association between microRNA-499 rs3746444 polymorphism and cancer risk of urinary system in allelic model. PC, prostate cancer; BLC, bladder cancer; RCC, renal cell cancer.

**Figure 5:**
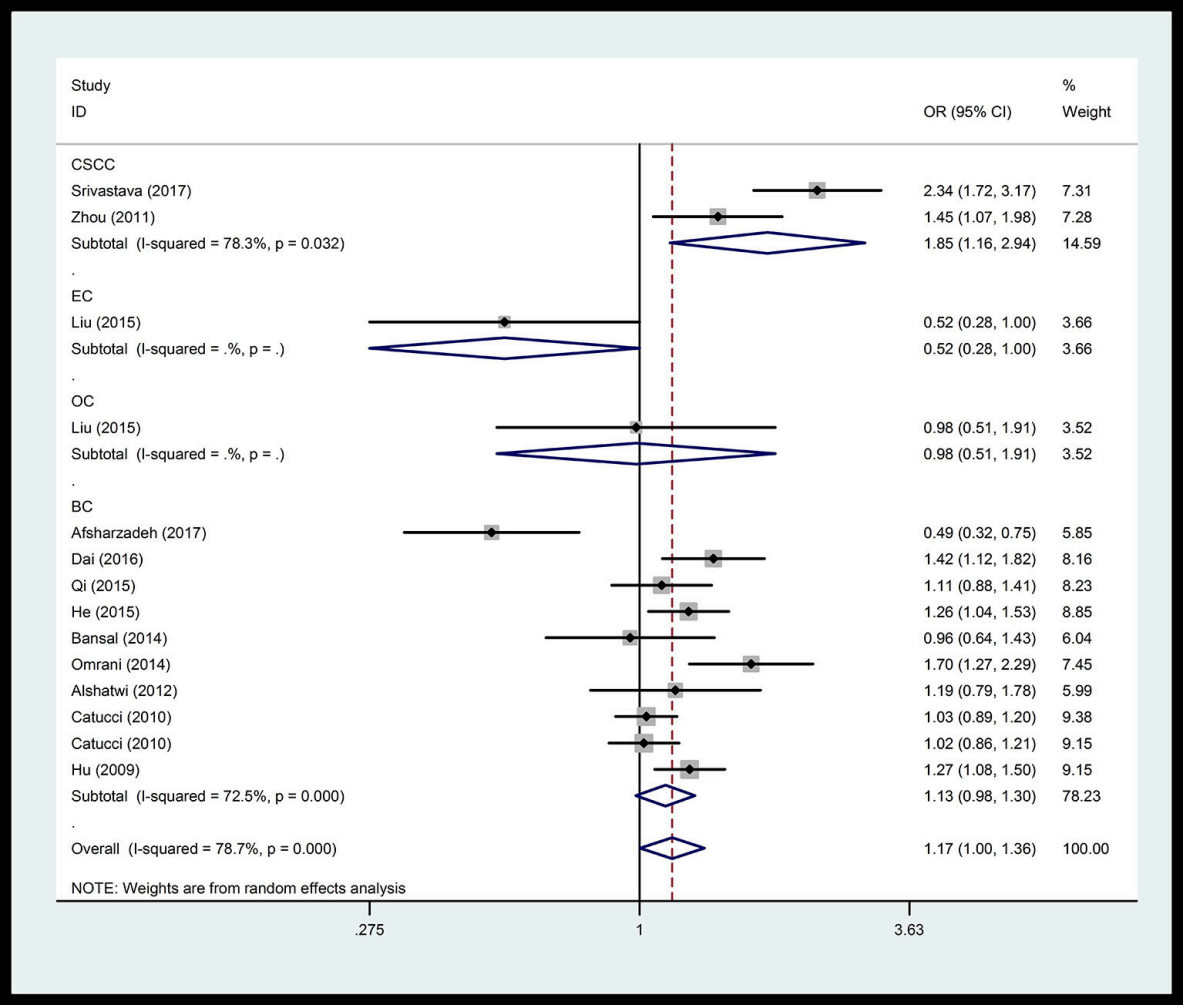
Forest plot of the OR and 95% CIs for association between microRNA-499 rs3746444 polymorphism and cancer risk of gynecological system in allelic model. BC, breast cancer; CSCC, cervical squamous cell carcinoma; EC, endometrial cancer; OC, ovarian cancer.

In the respiratory system, cancer risk was found to be significantly increased in the CC genetic model as compared with those in the TT and TC+TT (recessive) models (CC vs.TT: OR = 1.575, 95% CI = 1.268–1.955, *P* < 0.001; CC vs. TC+TT: OR = 1.527, 95% CI = 1.232–1.892, *P* < 0.001) (Figure 2), especially in lung cancer. We also found significantly increased cancer risk in all five genetic models of the digestive system (CC vs.TT: OR = 1.153, 95% CI = 1.027–1.295, *P* = 0.016; TC vs. TT: OR = 1.109, 95% CI = 1.046–1.176, *P* = 0.001; CC+TC vs. TT: OR = 1.112, 95% CI = 1.018–1.216, *P* = 0.019; CC vs. TC+TT: OR = 1.137, 95% CI = 1.016–1.272, *P* = 0.025; C vs. T: OR = 1.112, 95% CI = 1.025–1.206, *P* = 0.011; Figure 3), particularly in hepatocellular carcinoma. Significant association was observed between rs3746444 and increased cancer risk in three genetic models in the urinary system (TC vs. TT: OR = 1.307, 95% CI = 1.130–1.512, *P* < 0.001; CC+TC vs. TT = 1.259, 95% CI = 1.097–1.446, *P* = 0.001; C vs. T: OR = 1.132, 95% CI = 1.014–1.264, *P* = 0.027; Figure 4), as well as for prostate cancer. Our results showed that rs3746444 could increase cancer risk of the gynecological system in the allelic model (C vs. T: OR = 1.169, 95% CI = 1.002–1.364, *P* = 0.047; Figure 5), particularly for cervical squamous cell carcinoma. However, studies on lymphatic and hematopoietic system cancers did not reveal any significant association between cancer risk and genetic models. In addition, no significant association between the five different genetic models and cancer risk in other types of cancers were observed.

We then performed subgroup analysis based on ethnicity, source of control, and HWE in controls. When results were stratified by ethnicity, significant association was found between miR-499 rs3746444 polymorphism and cancer risk in the Asians; (CC vs. TT: OR = 1.301, 95% CI = 1.180–1.434, *P* < 0.001; TC vs. TT: OR = 1.123, 95% CI = 1.040–1.213, *P* = 0.003; CC+TC vs. TT: OR = 1.152, 95% CI = 1.065–1.247, *P* < 0.001; CC vs. TC+TT: OR = 1.267, 95% CI = 1.151–1.394, *P* < 0.001; C vs. T: OR = 1.136, 95% CI = 1.072–1.237, *P* < 0.001) this association was absent in the Caucasians. When analysis was performed on source of control, the ORs were significant in four genetic models for the population-based control. At the same time, significant associations were observed for the hospital-based control in the homozygote comparison model and the recessive model. Following the removal of 21 studies whose genotypic distributions in controls were not in accordance with HWE, the results remained the same.

### Sensitivity analysis

Sensitivity analysis was used to estimate the contribution of each study to the pooled ORs. Our results showed that the total ORs were not significantly altered following removal of studies where the controls were not in accordance with HWE. This further supported the significant association between miR-499 rs3746444 and cancer risk.

### Publication bias

**Figure 6:**
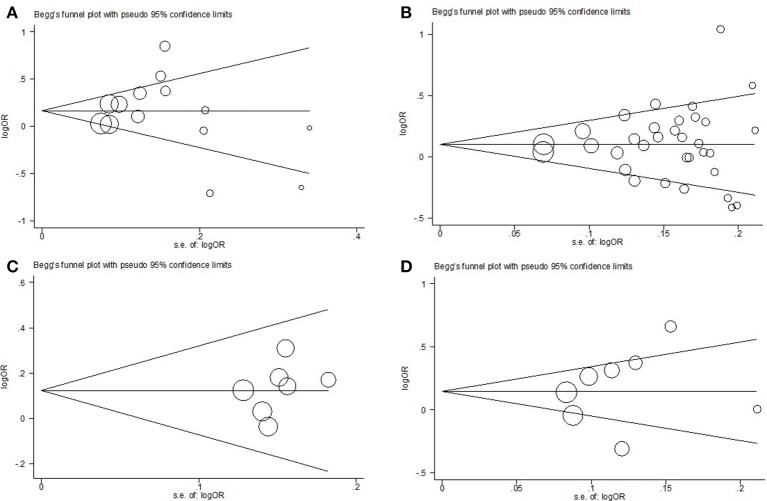
Funnel plot analysis to detect publication bias for allelic model. **(A)** respiratory system; **(B)** digestive system; **(C)** urinary system; **(D)** gynecological system.

In this meta-analysis, the potential effect of publication bias in literatures was estimated by funnel plots (Figure 6) and the Egger's test. Based on our analysis, no publication bias was found (*P* > 0.05).

## Discussion

The odds of getting cancer varies widely among different individuals. Many studies have discovered that miRNAs are strongly associated with tumorigenesis and can act as oncogenes or tumor suppressor genes that affect the initiation and development of tumors (Zhou et al., 2015). In recent years, the status of SNPs in microRNAs and their potential effects on cancer risk have been extensively studied. Though several studies and meta-analyses have been carried out to evaluate the association between miR-499 rs3746444 T > C polymorphism and cancer risk, no conclusive findings have been obtained in different cancers. Hence, integrating and updating these results to explore the relationship further is important.

In this study, our results demonstrate significant associations between miR-499 rs3746444 polymorphism and cancer susceptibility for the first time, in five different genetic models from 65 case-control studies. Meta-analyses of all types of cancer risks by other groups have found either no association between the miR-499 polymorphism and cancer susceptibility or significant associations in one or two genetic models (Xu et al., 2015). Several meta-analyses have explored the association between the miR-499 rs3746444 polymorphism and cancer risk and it is difficult to judge if the analysis without studies departing from HWE would be more valid or not. An overall meta-analysis by Jiang et al. (2015) demonstrated a borderline association (TC+CC vs. TT: OR = 1.15, *P* = 0.04). However, the association disappeared (TC+CC vs. TT: OR = 1.18, *P* = 0.21) when studies departing from HWE were excluded. In our study, after the exclusion of 21 studies wherein the genotypic distributions in controls were not in accordance with HWE, the results were still significant. Therefore, though there is still no consensus on whether or not to include studies that deviate from HWE, our results basically were not affected by such studies.

The most important feature of our study is that it is the first meta-analysis to explore the associations between miR-499 polymorphism and cancer susceptibility in different systems comprehensively. We found that microRNA-499 rs3746444 was significantly associated with an increased risk of cancer of the respiratory system in two genetic models, digestive system in all five genetic models, the urinary system in three genetic models, and gynecological system in an allelic model. To the best of our knowledge, no previous meta-analysis has explored this association in cancer of the respiratory system. We also found that miR-499 rs3746444 C allele might increase the risk of lung cancer in a homozygote comparison model. Previous studies have reported that rs3746444 was significantly associated with an increased risk of lung cancer and could contribute to a poor prognosis by modulating the expression of cancer-related genes leading to tumorigenesis and resistance to chemotherapy in lung cancer (Li D. et al., 2016). Our data differs from that of Li et al. (2013), since their analysis which included 12 gastrointestinal cancer studies, demonstrated no association between miR-499 rs3746444 and gastrointestinal cancer. We included 33 gastrointestinal cancer studies in this meta-analysis. In our analysis, though the number of the studies evaluating the association between hepatocellular carcinoma and miR-499 was the largest, the results were inconsistent on account of the small sample sizes and different ethnic groups. MicroRNA-499 downregulates the expression of the ets1 proto-oncogene in HepG2 cells and plays a vital role in the pathogenesis of HCC (Wei et al., 2012). In addition, we found that miR-499 rs3746444 C allele can increase the risk of hepatocellular carcinoma in recessive and allelic models. Our findings from the allelic hepatocellular carcinoma model were consistent with those of Yu et al. (2017). We also found that that rs3746444 could increase the risk of gynecological cancers in an allelic model. At the same time, miR-499 rs3746444 was associated with susceptibility to cervical squamous cell carcinoma. Consistent with the findings of Wang et al. (2017), we also found a significant association between rs3746444 and increased risk for cancer of the urinary system in two genetic models, but not the allelic model. Our results also suggested a significant association between miR-499 rs3746444 and prostate cancer risk in the heterozygote comparison and dominant models. George et al. have shown that a heterozygous miR-499 genotype, confers an increased risk of developing prostate cancer in the North Indian population (George et al., 2011). Nikolic et al. found that rs3746444 qualifies for a genetic variant potentially associated with aggressive prostate cancer in the Serbian population (Nikolic et al., 2015). Classifying cancer into different systems provides a new perspective and an in-depth understanding of the association between the miR-499 polymorphism and cancer susceptibility.

As for subgroup analysis based on ethnicity, our results were consistent with those of other meta-analyses. We found an association between the miR-499 polymorphism and cancer risk in Asians, but not in Caucasians. Unlike the other analyses, we found an association among the five genetic models in the Asian population. Our results suggest that miR-499 rs3746444 is associated with susceptibility to cervical squamous cell carcinoma, lung cancer, prostate cancer, and hepatocellular carcinoma, but not other cancer types, indicating that rs3746444 may have different effects on different cancer types. Compared to the previous studies, the difference in results could also be due to a lack of articles on miR-499 polymorphism and cancer risk.

Although our study pooled a large number of cases and controls including 52456 individuals, limitations which might affect the objectivity of the results still exist. First, our studies included data from only Asians and Caucasians and none from the African population. Second, detailed and original patient information such as age, gender, lifestyle, family history, nutrient intake was lacking. Third, gene-gene and gene-environment interactions should have been taken into consideration, if the relevant information was available.

In conclusion, our meta-analysis found that miR-499 rs3746444 polymorphism is associated with the risk of cancer in five genetic models. MicroRNA-499 rs3746444 was found to be significantly associated with increased risk of cancer of the respiratory, digestive, urinary, and gynecological systems. The subgroup analysis by ethnicity showed a significant association with increased cancer risk in the Asian population. Our findings also suggest that the miR-499 rs3746444 C allele may increase the risk of cervical squamous cell carcinoma, lung cancer, prostate cancer, and hepatocellular carcinoma. Well-designed and large-scale studies are, therefore, necessary to further verify these findings.

## Author contributions

XY and BZ designed this study. XY and XL searched the databases and extracted the data. XY wrote the manuscript. XL and BZ reviewed the manuscript. XY, XL, and BZ approved the final manuscript.

### Conflict of interest statement

The authors declare that the research was conducted in the absence of any commercial or financial relationships that could be construed as a potential conflict of interest.

## References

[B1] Afsharzadeh S. M. Mohaddes Ardebili S. M. Seyedi S. M. Karimian Fathi N. Mojarrad M. (2017). Association between rs11614913, rs3746444, rs2910164 and occurrence of breast cancer in Iranian population. Meta Gene 11, 20–25. 10.1016/j.mgene.2016.11.004

[B2] Ahn D. H. Rah H. Choi Y. K. Jeon Y. J. Min K. T. Kwack K.. (2013). Association of the miR-146aC>G, miR-149T>C, miR-196a2T>C, and miR-499A>G polymorphisms with gastric cancer risk and survival in the Korean population. Mol. Carcinog. 52(Suppl. 1), E39–E51. 10.1002/mc.21962 23001871

[B3] Akkiz H. Bayram S. Bekar A. Akgollu E. Uskudar O. (2011). Genetic variation in the microRNA-499 gene and hepatocellular carcinoma risk in a Turkish population: lack of any association in a case-control study. Asian Pac. J. Cancer Prev. 12, 3107–3112. 22393998

[B4] Alshatwi A. A. Shafi G. Hasan T. N. Syed N. A. Al-Hazzani A. A. Alsaif M. A.. (2012). Differential expression profile and genetic variants of microRNAs sequences in breast cancer patients. PLoS ONE 7:e30049. 10.1371/journal.pone.0030049 22363415 PMC3282723

[B5] Ando H. Asai T. Koide H. Okamoto A. Maeda N. Tomita K.. (2014). Advanced cancer therapy by integrative antitumor actions via systemic administration of miR-499. J. Control. Release 181, 32–39. 10.1016/j.jconrel.2014.02.019 24593893

[B6] Bansal C. Sharma K. L. Misra S. Srivastava A. N. Mittal B. Singh U. S. (2014). Common genetic variants in pre-microRNAs and risk of breast cancer in the North Indian population. Ecancermedicalscience 8:473. 10.3332/ecancer.2014.473 25374621 PMC4208924

[B7] Bartel D. P. (2004). MicroRNAs: genomics, biogenesis, mechanism, and function. Cell 116, 281–297. 10.1016/S0092-8674(04)00045-5 14744438

[B8] Cai M. Zhang Y. Ma Y. Li W. Min P. Qiu J.. (2015). Association between microRNA-499 polymorphism and gastric cancer risk in Chinese population. Bull. Cancer 102, 973–978. 10.1016/j.bulcan.2015.09.012 26597478

[B9] Catucci I. Yang R. Verderio P. Pizzamiglio S. Heesen L. Hemminki K.. (2010). Evaluation of SNPs in miR-146a, miR196a2 and miR-499 as low-penetrance alleles in German and Italian familial breast cancer cases. Hum. Mutat. 31, E1052–E1057. 10.1002/humu.21141 19847796

[B10] Chu Y. H. Hsieh M. J. Chiou H. L. Liou Y. S. Yang C. C. Yang S. F.. (2014). MicroRNA gene polymorphisms and environmental factors increase patient susceptibility to hepatocellular carcinoma. PLoS ONE 9:e89930. 10.1371/journal.pone.0089930 24587132 PMC3935960

[B11] Dai Z. M. Kang H. F. Zhang W. G. Li H. B. Zhang S. Q. Ma X. B.. (2016). The associations of single nucleotide polymorphisms in miR196a2, miR-499, and miR-608 with breast cancer susceptibility: a STROBE-compliant observational study. Medicine 95:e2826. 10.1097/MD.0000000000002826 26886638 PMC4998638

[B12] Deng S. Wang W. Li X. Zhang P. (2015). Common genetic polymorphisms in pre-microRNAs and risk of bladder cancer. World J. Surg. Oncol. 13:297 10.1186/s12957-015-0683-6 26458899 PMC4603775

[B13] Dikaiakos P. Gazouli M. Rizos S. Zografos G. Theodoropoulos G. E. (2015). Evaluation of genetic variants in miRNAs in patients with colorectal cancer. Cancer Biomark. 15, 157–162. 10.3233/CBM-140449 25519012 PMC12928517

[B14] Du M. Lu D. Wang Q. Chu H. Tong N. Pan X.. (2014). Genetic variations in microRNAs and the risk and survival of renal cell cancer. Carcinogenesis 35, 1629–1635. 10.1093/carcin/bgu082 24681820

[B15] George G. P. Gangwar R. Mandal R. K. Sankhwar S. N. Mittal R. D. (2011). Genetic variation in microRNA genes and prostate cancer risk in North Indian population. Mol. Biol. Rep. 38, 1609–1615. 10.1007/s11033-010-0270-4 20842445

[B16] Gutierrez-Camino A. Lopez-Lopez E. Martin-Guerrero I. Pinan M. A. Garcia-Miguel P. Sanchez-Toledo J.. (2014). Noncoding RNA-related polymorphisms in pediatric acute lymphoblastic leukemia susceptibility. Pediatr. Res. 75, 767–773. 10.1038/pr.2014.43 24618566

[B17] Hao Y. X. Wang J. P. Zhao L. F. (2014). Associations between three common MicroRNA polymorphisms and hepatocellular carcinoma risk in Chinese. Asian Pac. J. Cancer Prev. 14, 6601–6604. 10.7314/APJCP.2013.14.11.6601 24377574

[B18] Hasani S. S. Hashemi M. Eskandari-Nasab E. Naderi M. Omrani M. Sheybani-Nasab M. (2014). A functional polymorphism in the miR-146a gene is associated with the risk of childhood acute lymphoblastic leukemia: a preliminary report. Tumour Biol. 35, 219–225. 10.1007/s13277-013-1027-1 23888320

[B19] Hashemi M. Moradi N. Ziaee S. A. Narouie B. Soltani M. H. Rezaei M.. (2016). Association between single nucleotide polymorphism in miR-499, miR-196a2, miR-146a and miR-149 and prostate cancer risk in a sample of Iranian population. J. Adv. Res. 7, 491–498. 10.1016/j.jare.2016.03.008 27222754 PMC4856822

[B20] He B. Pan Y. Xu Y. Deng Q. Sun H. Gao T.. (2015). Associations of polymorphisms in microRNAs with female breast cancer risk in Chinese population. Tumour Biol. 36, 4575–4582. 10.1007/s13277-015-3102-2 25613069

[B21] Hou Y. Y. Lee J. H. Chen H. C. Yang C. M. Huang S. J. Liou H. H.. (2015). The association between miR-499a polymorphism and oral squamous cell carcinoma progression. Oral Dis. 21, 195–206. 10.1111/odi.12241 24690080

[B22] Hu X. Li L. Shang M. Zhou J. Song X. Lu X.. (2014). Association between microRNA genetic variants and susceptibility to colorectal cancer in Chinese population. Tumour Biol. 35, 2151–2156. 10.1007/s13277-013-1285-y 24136745

[B23] Hu Z. Liang J. Wang Z. Tian T. Zhou X. Chen J.. (2009). Common genetic variants in pre-microRNAs were associated with increased risk of breast cancer in Chinese women. Hum. Mutat. 30, 79–84. 10.1002/humu.20837 18634034

[B24] Jiang S. G. Chen L. Tang J. H. Zhao J. H. Zhong S. L. (2015). Lack of association between Hsa-Mir-499 rs3746444 polymorphism and cancer risk: meta-analysis findings. Asian Pac. J. Cancer Prev. 16, 339–344. 10.7314/APJCP.2015.16.1.339 25640376

[B25] Jovanovic M. Hengartner M. O. (2006). miRNAs and apoptosis: RNAs to die for. Oncogene 25, 6176–6187. 10.1038/sj.onc.1209912 17028597

[B26] Kim W. H. Min K. T. Jeon Y. J. Kwon C. I. Ko K. H. Park P. W.. (2012). Association study of microRNA polymorphisms with hepatocellular carcinoma in Korean population. Gene 504, 92–97. 10.1016/j.gene.2012.05.014 22583825

[B27] Kou J. T. Fan H. Han D. Li L. Li P. Zhu J.. (2014). Association between four common microRNA polymorphisms and the risk of hepatocellular carcinoma and HBV infection. Oncol. Lett. 8, 1255–1260. 10.3892/ol.2014.2257 25120701 PMC4114578

[B28] Li D. Peng J. J. Tan Y. Chen T. Wei D. Du M.. (2015). Genetic variations in microRNA genes and susceptibility to hepatocellular carcinoma. Genet. Mol. Res. 14, 1926–1931. 10.4238/2015 25867338

[B29] Li D. Zhu G. Di H. Li H. Liu X. Zhao M.. (2016). Associations between genetic variants located in mature microRNAs and risk of lung cancer. Oncotarget 7, 41715–41724. 10.18632/oncotarget.9566 27232940 PMC5173090

[B30] Li L. Sheng Y. Lv L. Gao J. (2013). The association between Two MicroRNA variants (miR-499, miR-149) and gastrointestinal cancer risk: a meta-analysis. PLoS ONE 8:e81967. 10.1371/journal.pone.0081967 24312386 PMC3843688

[B31] Li M. Zhang S. Wu N. Wu L. Wang C. Lin Y. (2016). Overexpression of miR-499-5p inhibits non-small cell lung cancer proliferation and metastasis by targeting VAV3. Sci. Rep. 6:23100. 10.1038/srep23100 26972445 PMC4789784

[B32] Li X. Li K. Wu Z. (2015). Association of four common SNPs in microRNA polymorphisms with the risk of hepatocellular carcinoma. Int. J. Clin. Exp. Pathol. 8, 9560–9566. 26464719 PMC4583951

[B33] Liu X. Xu B. Li S. Zhang B. Mao P. Qian B.. (2015). Association of SNPs in miR-146a, miR-196a2, and miR-499 with the risk of endometrial/ovarian cancer. Acta Biochim. Biophys. Sin. 47, 564–566. 10.1093/abbs/gmv042 26008204

[B34] Liu X. Zhang Z. Sun L. Chai N. Tang S. Jin J.. (2011). MicroRNA-499-5p promotes cellular invasion and tumor metastasis in colorectal cancer by targeting FOXO4 and PDCD4. Carcinogenesis 32, 1798–1805. 10.1093/carcin/bgr213 21934092

[B35] Liu Z. Li G. Wei S. Niu J. El-Naggar A. K. Sturgis E. M.. (2010). Genetic variants in selected pre-microrna genes and the risk of squamous cell carcinoma of the head and neck. Cancer 116, 4753–4760. 10.1002/cncr.25323 20549817 PMC3030480

[B36] Ma Y. Wang R. Zhang J. Li W. Gao C. Liu J.. (2014). Identification of miR-423 and miR-499 polymorphisms on affecting the risk of hepatocellular carcinoma in a large-scale population. Genet. Test. Mol. Biomarkers 18, 516–524. 10.1089/gtmb.2013.0510 24854593 PMC4094005

[B37] Min K. T. Kim J. W. Jeon Y. J. Jang M. J. Chong S. Y. Oh D.. (2012). Association of the miR-146aC>G, 149C>T, 196a2C>T, and 499A>G polymorphisms with colorectal cancer in the Korean population. Mol. Carcinog. 51(Suppl. 1), E65–E73. 10.1002/mc.21849 22161766

[B38] Mittal R. D. Gangwar R. George G. P. Mittal T. Kapoor R. (2011). Investigative role of pre-microRNAs in bladder cancer patients: a case-control study in North India. DNA Cell Biol. 30, 401–406. 10.1089/dna.2010.1159 21345130

[B39] Nikolic Z. Savic Pavicevic D. Vucic N. Cidilko S. Filipovic N. Cerovic S.. (2015). Assessment of association between genetic variants in microRNA genes hsa-miR-499, hsa-miR-196a2 and hsa-miR-27a and prostate cancer risk in Serbian population. Exp. Mol. Pathol. 99, 145–150. 10.1016/j.yexmp.2015.06.009 26112096

[B40] Okamoto A. Asai T. Ryu S. Ando H. Maeda N. Dewa T.. (2016). Enhanced efficacy of doxorubicin by microRNA-499-mediated improvement of tumor blood flow. J. Clin. Med. 5:10. 10.3390/jcm5010010 26797645 PMC4730135

[B41] Okubo M. Tahara T. Shibata T. Yamashita H. Nakamura M. Yoshioka D.. (2010). Association between common genetic variants in Pre-microRNAs and gastric cancer risk in japanese population. Helicobacter 15, 524–531. 10.1111/j.1523-5378.2010.00806.x 21073609

[B42] Omrani M. Hashemi M. Eskandari-Nasab E. Hasani S. S. Mashhadi M. A. Arbabi F.. (2014). hsa-mir-499 rs3746444 gene polymorphism is associated with susceptibility to breast cancer in an Iranian population. Biomark. Med. 8, 259–267. 10.2217/bmm.13.118 24521023

[B43] Poltronieri-Oliveira A. B. Madeira F. F. Nunes D. B. S. M. Rodrigues G. H. Lopes B. C. Manoel-Caetano F. S. (2017). Polymorphisms of miR-196a2 (rs11614913) and miR-605 (rs2043556) confer susceptibility to gastric cancer. Gene Rep. 7, 154–163. 10.1016/j.genrep.2017.04.006

[B44] Pu J. Y. Dong W. Zhang L. Liang W. B. Yang Y. Lv M. L. (2014). No association between single nucleotide polymorphisms in pre-mirnas and the risk of gastric cancer in Chinese population. Iran. J. Basic Med. Sci. 17, 128–133. 10.22038/ijbms.2014.2246 24711897 PMC3976751

[B45] Qi J. H. Wang J. Chen J. Shen F. Huang J. T. Sen S. (2014). High-resolution melting analysis reveals genetic polymorphisms in microRNAs confer hepatocellular carcinoma risk in Chinese patients. BMC Cancer 14:643 10.1186/1471-2407-14-643 25176041 PMC4161871

[B46] Qi P. Wang L. Zhou B. Yao W. J. Xu S. Zhou Y.. (2015). Associations of miRNA polymorphisms and expression levels with breast cancer risk in the Chinese population. Genet. Mol. Res. 14, 6289–6296. 10.4238/2015.June.11.2 26125831

[B47] Qiu F. Yang L. Zhang L. Yang X. Yang R. Fang W.. (2015). Polymorphism in mature microRNA-608 sequence is associated with an increased risk of nasopharyngeal carcinoma. Gene 565, 180–186. 10.1016/j.gene.2015.04.008 25861865

[B48] Rogoveanu I. Burada F. Cucu M. G. Vere C. C. Ioana M. Cimpeanu R. A. (2017). Association of microRNA polymorphisms with the risk of gastric cancer in a romanian population. J. Gastrointestin. Liver Dis. 26, 231–238. 10.15403/jgld.2014.1121.263.rog 28922434

[B49] Shan Y. F. Huang Y. H. Chen Z. K. Huang K. T. Zhou M. T. Shi H. Q.. (2013). miR-499A>G rs3746444 and miR-146aG>C expression and hepatocellular carcinoma risk in the Chinese population. Genet. Mol. Res. 12, 5365–5371. 10.4238/2013.November.7.11 24301908

[B50] Shen F. Chen J. Guo S. Zhou Y. Zheng Y. Yang Y.. (2016). Genetic variants in miR-196a2 and miR-499 are associated with susceptibility to esophageal squamous cell carcinoma in Chinese Han population. Tumour Biol. 37, 4777–4784. 10.1007/s13277-015-4268-3 26518769

[B51] Srivastava K. Srivastava A. Mittal B. (2010). Common genetic variants in pre-microRNAs and risk of gallbladder cancer in North Indian population. J. Hum. Genet. 55, 495–499. 10.1038/jhg.2010.54 20520619

[B52] Srivastava S. Singh S. Fatima N. Mittal B. Srivastava A. N. (2017). Pre-microrna gene polymorphisms and risk of cervical squamous cell carcinoma. J. Clin. Diagnos. Res. 11, GC01–GC04. 10.7860/JCDR/2017/25361.10543 29207732 PMC5713754

[B53] Tian T. Shu Y. Chen J. Hu Z. Xu L. Jin G.. (2009). A functional genetic variant in microRNA-196a2 is associated with increased susceptibility of lung cancer in Chinese. Cancer Epidemiol. Biomarkers Prev. 18, 1183–1187. 10.1158/1055-9965.EPI-08-0814 19293314

[B54] Torre L. A. Bray F. Siegel R. L. Ferlay J. Lortet-Tieulent J. Jemal A. (2015). Global cancer statistics, 2012. CA Cancer J. Clin. 65, 87–108. 10.3322/caac.21262 25651787

[B55] Umar M. Upadhyay R. Prakash G. Kumar S. Ghoshal U. C. Mittal B. (2013). Evaluation of common genetic variants in pre-microRNA in susceptibility and prognosis of esophageal cancer. Mol. Carcinog. 52, 10–18. 10.1002/mc.21931 22692992

[B56] Vinci S. Gelmini S. Mancini I. Malentacchi F. Pazzagli M. Beltrami C.. (2013). Genetic and epigenetic factors in regulation of microRNA in colorectal cancers. Methods 59, 138–146. 10.1016/j.ymeth.2012.09.002 22989523

[B57] Vinci S. Gelmini S. Pratesi N. Conti S. Malentacchi F. Simi L.. (2011). Genetic variants in miR-146a, miR-149, miR-196a2, miR-499 and their influence on relative expression in lung cancers. Clin. Chem. Lab. Med. 49, 2073–2080. 10.1515/CCLM.2011.708 21902575

[B58] Wang J. Zhang Y. Zhang Y. Chen L. (2016). Correlation between miRNA-196a2 and miRNA-499 polymorphisms and bladder cancer. Int. J. Clin. Exp. Med. 9, 20484–20488.

[B59] Wang L. Zhang N. Pan H. P. Wang Z. Cao Z. Y. (2015). MiR-499-5p contributes to hepatic insulin resistance by suppressing PTEN. Cell. Physiol. Biochem. 36, 2357–2365. 10.1159/000430198 26279439

[B60] Wang X. H. Wang F. R. Tang Y. F. Zou H. Z. Zhao Y. Q. (2014). Association of miR-149C>T and miR-499A>G polymorphisms with the risk of hepatocellular carcinoma in the Chinese population. Genet. Mol. Res. 13, 5048–5054. 10.4238/2014.July.4.20 25061729

[B61] Wang Y. H. Hu H. N. Weng H. Chen H. Luo C. L. Ji J.. (2017). Association between polymorphisms in MicroRNAs and risk of urological cancer: a meta-analysis based on 17,019 subjects. Front. Physiol. 8:325. 10.3389/fphys.2017.00975 28579964 PMC5437731

[B62] Wei J. Zheng L. Liu S. Yin J. Wang L. Wang X.. (2013). MiR-196a2 rs11614913 T > C polymorphism and risk of esophageal cancer in a Chinese population. Hum. Immunol. 74, 1199–1205. 10.1016/j.humimm.2013.06.012 23792053

[B63] Wei W. Hu Z. Fu H. Tie Y. Zhang H. Wu Y.. (2012). MicroRNA-1 and microRNA-499 downregulate the expression of the ets1 proto-oncogene in HepG2 cells. Oncol. Rep. 28, 701–706. 10.3892/or.2012.1850 22664953

[B64] Wilson K. D. Hu S. Venkatasubrahmanyam S. Fu J. D. Sun N. Abilez O. J.. (2010). Dynamic microRNA expression programs during cardiac differentiation of human embryonic stem cells: role for miR-499. Circ. Cardiovasc. Genet. 3, 426–435. 10.1161/CIRCGENETICS.109.934281 20733065 PMC3057038

[B65] Wu X. J. Mi Y. Y. Yang H. Hu A. K. Li C. Li X. D.. (2013). Association of the hsa-mir-499 (rs3746444) polymorphisms with gastric cancer risk in the Chinese population. Onkologie 36, 573–576. 10.1159/000355518 24107911

[B66] Xiang Y. Fan S. Cao J. Huang S. Zhang L. P. (2012). Association of the microRNA-499 variants with susceptibility to hepatocellular carcinoma in a Chinese population. Mol. Biol. Rep. 39, 7019–7023. 10.1007/s11033-012-1532-0 22311030

[B67] Xu Z. Zhang E. Duan W. Sun C. Bai S. Tan X. (2015). The association between miR-499 polymorphism and cancer susceptibility: a meta-analysis. Onco. Targets. Ther. 8, 2179–2186. 10.2147/OTT.S88224 26347202 PMC4550183

[B68] Yan P. Xia M. Gao F. Tang G. Zeng H. Yang S.. (2015). Predictive role of miR-146a rs2910164 (C>G), miR-149 rs2292832 (T>C), miR-196a2 rs11614913 (T>C) and miR-499 rs3746444 (T>C) in the development of hepatocellular carcinoma. Int. J. Clin. Exp. Pathol. 8, 15177–15183. 26823863 PMC4713649

[B69] Ying H. Q. Peng H. X. He B. S. Pan Y. Q. Wang F. Sun H. L.. (2016). MiR-608, pre-miR-124-1 and pre-miR26a-1 polymorphisms modify susceptibility and recurrence-free survival in surgically resected CRC individuals. Oncotarget 7, 75865–75873. 10.18632/oncotarget.12422 27713147 PMC5342784

[B70] Yu J. Y. Hu F. Du W. Ma X. L. Yuan K. (2017). Study of the association between five polymorphisms and risk of hepatocellular carcinoma: a meta-analysis. J. Chin. Med. Assoc. 80, 191–203. 10.1016/j.jcma.2016.09.009 28188097

[B71] Zhang E. Xu Z. Duan W. Huang S. Lu L. (2017). Association between polymorphisms in pre-miRNA genes and risk of oral squamous cell cancer in a Chinese population. PLoS ONE 12:e0176044. 10.1371/journal.pone.0176044 28609461 PMC5469449

[B72] Zhang L. H. Hao B. B. Zhang C. Y. Dai X. Z. Zhang F. (2016). Contributions of polymorphisms in miR146a, miR196a, and miR499 to the development of hepatocellular carcinoma. Genet. Mol. Res. 15:gmr.15038582. 10.4238/gmr.15038582 27706712

[B73] Zhang L. L. Liu J. J. Liu F. Liu W. H. Wang Y. S. Zhu B.. (2012). MiR-499 induces cardiac differentiation of rat mesenchymal stem cells through wnt/beta-catenin signaling pathway. Biochem. Biophys. Res. Commun. 420, 875–881. 10.1016/j.bbrc.2012.03.092 22465011

[B74] Zhou B. Dong L. P. Jing X. Y. Li J. S. Yang S. J. Wang J. P.. (2014). Association between miR-146aG>C and miR-196a2C>T polymorphisms and the risk of hepatocellular carcinoma in a Chinese population. Tumour Biol. 35, 7775–7780. 10.1007/s13277-014-2020-z 24816919

[B75] Zhou B. Wang K. Wang Y. Xi M. Zhang Z. Song Y.. (2011). Common genetic polymorphisms in pre-microRNAs and risk of cervical squamous cell carcinoma. Mol. Carcinog. 50, 499–505. 10.1002/mc.20740 21319225

[B76] Zhou J. Lv R. Song X. Li D. Hu X. Ying B.. (2012). Association between two genetic variants in miRNA and primary liver cancer risk in the Chinese population. DNA Cell Biol. 31, 524–530. 10.1089/dna.2011.1340 21861697 PMC3322400

[B77] Zhou X. Du Y. L. Jin P. Ma F. (2015). Bioinformatic analysis of cancer-related microRNAs and their target genes. Yi Chuan 37, 855–864. 10.16288/j.yczz.14-439 26399525

[B78] Zou H. Z. Zhao Y. Q. (2013). Positive association between miR-499A>G and hepatocellular carcinoma risk in a Chinese population. Asian Pac. J. Cancer Prev. 14, 1769–1772. 23679271 10.7314/apjcp.2013.14.3.1769

